# Effects of pregnant women’s body image on their sexual attitudes

**DOI:** 10.1186/s12884-026-08876-x

**Published:** 2026-03-03

**Authors:** Mehtap Genç, Yasemin Şanlı

**Affiliations:** 1 Department of Mental Health and Psychiatric Nursing, Faculty of Health Sciences, Karamanoğlu Mehmetbey University, Karaman, 70100 Türkiye; 2 Department of Midwifery, Faculty of Health Sciences, Karamanoğlu Mehmetbey University, Karaman, Türkiye

**Keywords:** Body perception, Sexual attitude, Pregnancy

## Abstract

In this descriptive, correlational study, the aim was to investigate the effect of body image on sexual attitudes in pregnant women. The study was conducted with 460 pregnant women who attended the Pregnancy School of a Training and Research Hospital in Turkey between March 2025 and July 2025. Data were collected using the Descriptive Characteristics Form, Body Understanding Measure for Pregnancy Scale (BUMPS), and Attitudes towards Sexuality in Pregnancy Scale (ASPS). Pearson correlation was used to analyze the relationships between continuous variables, and the multiple linear regression analysis was used to determine the factors affecting attitudes towards sexuality using backward elimination. The mean age of the participants was 28.42 ± 5.04 years. The largest proportion held a bachelor’s degree, and a substantial proportion of their spouses also had a bachelor’s degree. The mean score the participants obtained from the ASPS was 123.50 ± 20.06, above the cut-off point (111.5); indicating that the *participating pregnant women’s* (hereafter referred to as “participants”) attitudes towards sexuality were generally positive. The mean score they obtained from the BUMPS score was 50.91 ± 11.87, indicating that they had a moderately positive body image. The results of the multiple linear regression analysis revealed that the educational status of the participants and their partners, and certain pre-pregnancy and pregnancy-related factors significantly affected the participants’ attitudes towards sexuality during pregnancy. In conclusion, as the participants’ acceptance of their body image increased and their concerns about weight gain decreased, they displayed more positive attitudes towards sexuality. It is recommended that nurses should be encouraged to plan supportive interventions that address pregnant women’s body image and attitudes towards sexuality.

**Supplementary Information:**

The online version contains supplementary material available at 10.1186/s12884-026-08876-x.

## Background

Body image, a person’s internalized self-perception of his or her own body, is multidimensional. Body image encompasses a person’s perceptions, thoughts, attitudes, and feelings regarding his or her physical characteristics such as weight, shape, thinness, muscularity, sexual attractiveness, athletic appearance, and functionality [1]. During pregnancy, the body’s shape, weight, and resulting appearance change, causing the pregnant woman to focus on her own body. Although pregnancy is a normal physiological process, women can experience both physical and psychological problems during this period due to changes in their body image [2].

Changes in a pregnant woman’s body begin in the first trimester, but they don’t affect their body image, because they develop slowly. As pregnancy progresses, these changes can affect women both positively and negatively. Rapid and intense changes occur in a pregnant woman’s body during the second trimester, and these changes can cause the woman to have a different perception of herself and to feel clumsy, awkward, ugly, or unattractive. Due to these changes, a pregnant woman’s self-confidence and self-esteem can decrease, which, in turn, negatively affects her body image [3]. In the third trimester, these changes reach their peak. Due to the changes experienced throughout pregnancy, some pregnant women perceive their body image positively, while others are dissatisfied with it. In a study in which pregnant women’s self-esteem and body image were compared, their self-esteem was better in the first trimester than was that in the second and third trimesters [4].

Among the factors that influence body image during pregnancy are readiness for motherhood, planned pregnancy, adequate knowledge about pregnancy and birth, socioeconomic conditions, and a woman’s perception of her physical characteristics. In their study Küçükkaya et al. [5] determined that as the woman’ s pregnancy-related body image improved, so did her acceptance of pregnancy, and that her improved acceptance of pregnancy had a positive effect on her body image [5]. While some women experience a more comfortable pregnancy without any complications, others experience a more stressful pregnancy. The stress experienced due to weight gain and changes in appearance can cause pregnant women to have a sense of inadequacy and various emotional problems that can lead to crises. This feeling of inadequacy can cause pregnant women to perceive their body image negatively [6]. As a result of having this negative perception of body image, many pregnant women may strive to maintain their pre-pregnancy body standards. They may eat unhealthily out of concern for weight gain, leading to significant health problems for both themselves and their babies.

During pregnancy, body image directly affects a woman’s attitudes and behaviors towards pregnancy and sexuality. Among pregnancy-related changes are nausea and vomiting, worsened sleep and excretion patterns, breast swelling and tenderness, and skin changes like striae gravidarum and linea nigra, which women struggle to cope with and adapt to. Pregnant women may feel overweight and worry about whether they will regain their former appearance after giving birth. All these factors, in turn, affect a woman’s sexual life [7, 8].

Women’s sexual functioning, and attitudes towards sexuality vary throughout their married lives, especially during pregnancy. During pregnancy, women and their partners experience a dilemma: on the one hand, they postpone sexual intercourse out of fear of harming the baby, and on the other hand, they consider that sexual intercourse is necessary to maintain a healthy marriage [9, 10]. In a study, 88.8% of the participating pregnant women experienced sexual dysfunction, and the rates of sexual dysfunction in the first, second and third trimester were 81.8%, 85.7%, and 91.9%, respectively [11]. In Nakic Rados et al.’s study [12], 79.3% of the women experienced a decrease in the frequency of sexual intercourse compared to their pre-pregnancy period, 41% of them did not engage in sexual intercourse in the last month of pregnancy, and their sexual desire and sexual function decreased in the third trimester [12]. In Jamali and Mosalanejad’s study conducted in Iran (2013), 76.2% of the participating pregnant women experienced sexual dysfunction in the third trimester [13]. As determined in several other studies, factors such as back pain, weight gain, and dyspnea during the third trimester of pregnancy led to abstinence from sexual activities [12, 14]. As determined in many studies, pregnant women’s sexuality-related anxiety, beliefs, and values, and their attitudes towards approving sexuality during pregnancy affect their sexual functioning [12, 14–17].

Displaying a positive attitude towards sexuality during pregnancy can reduce sexual dysfunction [18]. In Erbil’s study (2019), it was determined that being overweight and obese negatively affected pregnant women’s sexual function, that the pregnant woman’s body image had no effect on sexual function, and that duration of pregnancy, number of pregnancies, fewer sexual intercourses, and changes in the partner’s sexual attitudes affected sexual function during pregnancy [11]. In Kumcağız’s study (2012), younger pregnant women had more positive body image than did other pregnant women, but they also exhibited negative attitudes towards sexuality during pregnancy [6]. In another study, higher education levels decreased women’s negative attitudes towards sexuality during pregnancy [10]. Pregnant women’s and their partners’ negative attitudes towards the physical and psychological changes that occur during pregnancy and their fear of harming the baby discourage them from having sexual intercourse during pregnancy [19, 20].

Since women experience physical, psychological, and sociocultural changes during pregnancy, the effect of their attitudes towards sexual function and sexuality during this period should not be overlooked. Being aware that pregnant women are likely to have negative body image and attitudes towards sexuality can help prevent them from experiencing potential psychological and sexual health problems during this period. Maintaining sexual functions during pregnancy, which affects physical, psychological, sociocultural, and family health, is of great importance in terms of protecting family health. The review of the literature revealed that in no studies, effects of pregnant women’s body image on their sexual attitudes were investigated.

Thus, in the current study, answers to the following questions were sought:

1. What is the body image of pregnant women like?
2. What attitudes do pregnant women display towards sexuality?
3. Does the body image of pregnant women affect their attitudes towards sexuality?
4. What are the factors that influence pregnant women’s body image and attitudes towards sexuality?

## Method

### Design and participants

In the present study, the descriptive and correlational research design was used. The study population consisted of pregnant women who attended the Pregnancy School at a Training and Research Hospital in Turkey. Based on hospital records from the previous year (2024), the known population size was 983 pregnant women. The minimum sample size was calculated as 276 using the sampling method for known population (confidence interval: 95%, margin of error: 0.05). However, considering the possibility of withdrawals and/or losses during the study, it was decided to include a higher number of pregnant women (*N* = 460) in the sample.

### Inclusion criteria

Being ≥ 18 years old, volunteering to participate in the study, being fluent in Turkish, being literate, having no diagnosis of a chronic or psychiatric illness, not being diagnosed with a high-risk pregnancy, being primiparous or multiparous, and being in the second or third trimester of pregnancy.

## Research variables

Independent variables: socio-demographic and obstetric characteristics of the participants, and their body perception,

Dependent variable: Scores obtained from the Attitudes towards Sexuality in Pregnancy Scale (ASPS).

### Data collection

This single-center study was conducted at a Training and Research Hospital in Turkey. Data were collected from the pregnant women who attended the Pregnancy School between March 2025 and July 2025 after the necessary permissions were obtained. After the women were informed about the purpose and content of the study in collaboration with the hospital, written informed consent was obtained from those who met the inclusion criteria and volunteered to participate. Then, data were collected from them through face-to-face interviews, conducted in a single session, in a quiet, confidential environment, which ensured that the interviews were conducted comfortably, safely, and effectively.

### Data collection tools

#### Descriptive characteristics form

The form consists of items questioning the participants’ socio-demographic characteristics such as age, education level, employment status, family type and income level, and obstetric characteristics such as desire for pregnancy, and health problems during pregnancy.

#### Body understanding measure for pregnancy scale (BUMPS)

The BUMPS was developed by Kirk and Preston (2019) [21]. The validity study of the Turkish version of the BUMPS was performed by Duman et al. (2023) [1]. The BUMPS administered to assess pregnant women’s body image during pregnancy consists of 19 items whose responses are rated on a five-point Likert-type scale ranging from 1 to 5, and the following 3 subscales: “Being Satisfied Appearing Pregnant”, “Concerns about Weight Gain”, “Physical Burdens of Pregnancy”. The minimum and maximum possible scores that can be obtained from the BUMPS are 19 and 95, respectively. The higher the score obtained from the “Acceptance of Physical Appearance of Pregnancy” subscale is, the lower the level of body satisfaction during pregnancy is. The higher the scores obtained from the “Concerns about Weight Gain” and “Physical Burdens of Pregnancy” subscales are, the higher the levels of negative perception and concern related to these areas are. The scale consists of positively and negatively keyed statements. While positively keyed statements express undesirable emotions, negatively keyed statements express desired emotions. Items 1, 4, 6, 8, 10, 11, 15, and 19 in the BUMPS are negatively keyed statements and the scores obtained from them are reversed. The scale has no cut-off point. The Cronbach’s α reliability coefficient of the BUMPS was calculated as 0.91 in Kirk & Preston’s, and Duman et al.’s studies, and 0.85 in the present study.

#### Attitudes towards sexuality in pregnancy scale (ASPS)

The ASPS developed by Yılmaz Sezer and Şentürk Erenel [10] is used to assess pregnant women’s and their partners’ attitudes towards sexuality during pregnancy. The ASPS consists of 34 items and the following 3 subscales: Anxiety towards Sexual Intercourse during Pregnancy (items 7, 10, 15, 18, 22, 25, 26, 27, 30), Beliefs and Values ​​Towards Sexuality during Pregnancy (items 3, 4, 8, 9, 12, 13, 16, 17, 19, 29), and Approval of Sexuality during Pregnancy (items 1, 2, 5, 6, 11, 14, 20, 21, 23, 24, 28, 31, 32, 33, 34). Items 1, 2, 5, 6, 11, 14, 20, 21, 23, 24, 28, 31, 32, 33, 34 refer to positive attitudes. Items 3, 4, 7, 8, 9, 10, 12, 13, 15, 16, 17, 18, 19, 22, 25, 26, 27, 29, 30 which refer to negative attitudes are reverse scored. The lowest and highest possible scores that can be obtained from the ASPS are 34 and 170, respectively. While the lowest possible scores that can be obtained from the “Anxiety towards Sexual Intercourse during Pregnancy”, “Beliefs and Values ​​Towards Sexuality during Pregnancy” and Approval of Sexuality during Pregnancy subscales are 9, 10, and 15, respectively, the highest possible scores that can be obtained from them are 45, 50, and 75, respectively. The higher the score obtained from the ASPS is, the more positive the respondent’s attitude towards sexuality during pregnancy is. The cut-off point for the ASPS is 111.5. If the score is above the cut-off point, it indicates positive attitude, and if it is below the cut-off point, it indicates negative attitude. The Cronbach’s Alpha coefficients of the overall ASPS and its “Anxiety towards Sexual Intercourse”, “Beliefs and Values ​​towards Sexuality during Pregnancy” and “Approval of Sexuality during Pregnancy” subscales were 0.90, 0.85, 0.86 and 0.81, respectively in Yılmaz Sezer and Şentürk Erenel’s study [10], and 0.92, 0.84, 0.90, 0.83, respectively in the present study.

### Statistical analysis

The data obtained were analyzed using the IBM SPSS (Statistical Package for the Social Sciences) version 23.0. Normality analysis was conducted to determine whether the properties of continuous variables were normally distributed. In this context, the Kolmogorov-Smirnov test was applied, and skewness and kurtosis values, histogram and Q-Q plot graphs, were taken into account. While continuous variables were presented as mean ± standard deviation (Mean ± SD), categorical variables were presented as number (n) and percentage (%). Pearson’s correlation coefficient was calculated to analyze the relationship between the continuous variables. Multiple linear regression analysis based on the backward elimination method was performed to determine the factors affecting attitudes towards sexuality during pregnancy. p values less than 0.05 were considered significant.

### Ethical considerations

To conduct the study, ethics committee approval was obtained from the Karamanoğlu Mehmetbey University Health Sciences Scientific Research and Publication Ethics Committee (Decision Date: September 04. 2024, Decision Number: 01-2024/09). Permission to conduct the study in the aforementioned hospital was obtained from the Provincial Health Directorate. Participation in the study was voluntary, and written informed consent was obtained from all the pregnant women who volunteered to participate in the study.

## Results

The mean age of the participants and their spouses were 28.42 ± 5.04 (min: 18, max: 45) and 31.58 ± 5.42 (min: 20, max: 53) years, respectively. The mean duration of marriage was 5.02 ± 4.39 years (min: 1, max: 25). The mean number of pregnancies was 2.09 ± 1.20 (min: 1, max: 7). The mean number of living children was 0.81 ± 0.93 (min: 0, max: 4). The mean gestational age was 27.98 ± 8.41 (min: 13, max: 40) weeks. The mean age at first pregnancy was 24.52 ± 4.01 (min: 16, max: 40) years. The average pre-pregnancy weight of the participants was 63.89 ± 12.60 (min: 30, max: 110) kg. The average weight during pregnancy (current weight) was 73.35 ± 13.25 (min: 40, max: 124) kg. The average height was 161.85 ± 6.13 (min: 145, max: 183) cm. Of the participants, 73.2% were homemakers and 42.4% had a bachelor’s degree. Of their spouses, 38.8% had a bachelor’s degree and 50.5% were workers. Of the participants, 71.7% perceived their income level as medium, 90.7% had a nuclear family, 14.1% had experienced curettage, 18% had experienced a miscarriage, 94.1% conceived naturally, 72.4% had planned pregnancies, 80.9% paid attention to their pre-pregnancy appearance, 72.4% paid attention to their pre-pregnancy weight, 42% experienced health problems during pregnancy, 30% experienced nausea and vomiting, 5.9% experienced excessive weight gain, 2.8% experienced increased blood pressure levels, 3.3% experienced increased blood glucose levels, and 8% experienced other health problems (Table 1). The other individual and pregnancy-related characteristics of the participants are presented in Table 1.

**Table 1:** Participants’ individual and pregnancy-related characteristics (*n* = 460)

Variables	Number (*n*)	Percentage (%)
Participants’ Age (years) (Mean: 28.42 ± 5.04. Min-Max:18–45)		
Participants’ Spouses’ Age (years) (Mean: 31.58 ± 5.42. Min-Max:20–53)		
Duration of marriage (years) (Mean: 5.02 ± 4.39. Min-Max:1–25)		
Number of pregnancies (Mean: 2.09 ± 1.20. Min-Max:1–7)		
Number of living children (Mean: 0.81 ± 0.93. Min-Max:0–4)		
Gestational age (weeks) (Mean: 27.98 ± 8.41. Min-Max:13–40)		
Age at first pregnancy (Mean: 24.52 ± 4.01. Min-Max:16–40)		
Pre-pregnancy weight (kg) (Mean: 63.89 ± 12.60. Min-Max:30–110)		
Current weight (kg) (Mean: 73.35 ± 13.25. Min-Max:40–124)		
Height (cm) (Mean: 161.85 ± 6.13. Min-Max:145–183)		
Employment status		
Homemaker	337	73.2
Civil servant	79	17.2
Worker	40	8.7
Retired	4	0.9
Education		
Primary school	12	2.6
Junior high school	82	17.8
Senior high school	138	30.0
Bachelor’s degree	195	42.4
Master’s degree	33	7.2
Spouses’ education		
Primary school	15	3.3
Junior high school	84	18.3
Senior high school	156	33.9
Bachelor’s degree	179	38.8
Master’s degree	26	5.7
Spouses’ employment status		
Civil servant	128	27.8
Worker	232	50.5
Self-employed	100	21.7
Perceived income		
Good	119	25.9
Medium	330	71.7
Bad	11	2.4
Family type		
Nuclear	417	90.7
Extended	43	9.3
Curettage		
Yes	65	14.1
No	395	85.9
Miscarriage		
Yes	83	18.0
No	377	82.0
Type of Pregnancy		
Naturally	433	94.1
Treatment	27	5.9
Whether the pregnancy was planned		
Planned	333	72.4
Unplanned	127	27.6
Paying attention to appearance before pregnancy		
Yes	372	80.9
No	88	19.1
Paying attention to weight before pregnancy		
Yes	333	72.4
No	127	27.6
Having health problems during pregnancy		
Yes	193	42.0
No	267	58.0
Nausea and vomiting		
Yes	138	30.0
No	322	70.0
Excessive weight gain		
Yes	27	5.9
No	433	94.1
Increase in blood pressure level		
Yes	13	2.8
No	447	97.2
Increase in blood glucose level		
Yes	15	3.3
No	445	96.7
Other health problems		
Yes	37	8.0
No	423	92.0
The way of perceiving current or potential changes in body image		
Positive	249	54.1
Negative	105	22.8
No idea	106	23.0
Being influenced by someone else in perceiving current or potential changes in body image		
Yes	54	11.7
No	406	88.3
Persons influencing their perception of body image		
Friends	4	7.4
Her family	13	24.1
Spouse	22	40.7
Spouse’s family	1	1.9
Social circle	14	25.9
The way how she was influenced		
Positively	31	57.4
Negatively	23	42.6
Being knowledgeable about having sexual intercourse during pregnancy		
Yes	370	80.4
No	90	19.6
Frequency of sexual intercourse before pregnancy		
Once a week	70	15.2
Twice a week	222	48.2
Three or more times a week	141	30.7
Once every two weeks	27	5.9
Being satisfied with sexual life before pregnancy		
No	13	2.8
Yes	447	97.2
Avoiding sexuality during pregnancy		
Yes	227	49.3
No	233	50.7

The mean score the participants obtained from the Attitudes towards Sexuality in Pregnancy Scale was 123.50 ± 20.06. Considering that the possible score range from the scale is 34–170 and the cut-off point is 111.5, the mean score the participants obtained was above the cut-off point (Table 2). The mean scores the participants obtained from the overall Body Understanding Measure for Pregnancy Scale and its “Acceptance of Physical Appearance of Pregnancy”, “Concerns about Weight Gain”, “Physical Burdens of Pregnancy” subscales were 50.91 ± 11.87, 24.76 ± 6.28, 17.63 ± 6.11 and 8.51 ± 2.43, respectively (Table 2).

**Table 2:** Mean scores the participants obtained from the scales

Scales	X	±SD	Min.	Max.	Possible score range	Cut-off point
Attitudes towards Sexuality in Pregnancy Scale	123.50	± 20.06	60	168	34–170	111.5
Body Understanding Measure for Pregnancy Scale	50.91	± 11.87	23	87	19–95	-
Acceptance of Physical Appearance of Pregnancy	24.76	± 6.28	9	41	9–45	-
Concerns about weight gain	17.63	± 6.11	7	35	7–35	-
Physical burdens of pregnancy	8.51	± 2.43	3	15	3–15	-

A low-level negative significant correlation was determined between the scores obtained from the “positive items of the Attitudes towards Sexuality in Pregnancy Scale” and the “Acceptance of Physical Appearance of Pregnancy” (*r*=-0.297, *p* < 0.001), “Concerns about Weight Gain” (*r*=-0.287, *p* < 0.001) and “Physical Burdens of Pregnancy” (*r*=-0.212, *p* < 0.001) subscales (Table 3).

**Table 3:** The relationship between the “Attitudes towards Sexuality in Pregnancy Scale”, and “Acceptance of Physical Appearance of Pregnancy”, “Concerns about Weight Gain” and. “Physical Burdens of Pregnancy subscales” (*n* = 460)

Variables		Attitudes towards Sexuality in Pregnancy Scale
Acceptance of Physical Appearance of Pregnancy	r	-0.297^**^
	p	0.000
Concerns about Weight Gain	r	-0.287^**^
	p	0.000
Physical Burdens of Pregnancy	r	-0.212^**^
	p	0.000

r: Pearson correlation coefficient, *: *p* < 0.05, **: *p* < 0.001

In Table 4, the results of the multivariate linear regression analysis conducted to determine the factors influencing attitudes towards sexuality during pregnancy are given. The created model significantly explained attitudes towards sexuality during pregnancy by 33.3% (F (11, 448) = 21.831, *p* < 0.001, Adj. R² = 0.333). The mean scores the participants with Bachelor’s and Master’s degrees obtained from the positive items of the Attitudes towards Sexuality in Pregnancy Scale were 6.856 and 13.812 units higher than were those obtained by the participants who were primary school graduates, respectively (*p* < 0.001). The mean score the participants whose spouses had a Bachelor’s degree obtained from the positive items of the Attitudes towards Sexuality in Pregnancy Scale was 4.177 units higher than was that obtained by the participants whose spouses were primary school graduates (*p* = 0.019). The mean score the participants who did not pay attention to their appearance before pregnancy obtained from the positive items of the Attitudes towards Sexuality in Pregnancy Scale was 4.818 units lower than was that obtained by the participants who paid attention to their appearance before pregnancy (*p* = 0.017). The mean score the participants who were not knowledgeable about sexuality during pregnancy obtained from the positive items of the Attitudes towards Sexuality in Pregnancy Scale was 10.395 units lower than was that obtained by the participants who were not knowledgeable (*p* < 0.001). The mean score the participants who were satisfied with their sexual life before pregnancy obtained from the positive items of the Attitudes towards Sexuality in Pregnancy Scale was 11.094 units higher than was that obtained by the participants who were not (*p* = 0.019). The mean score the participants who had sexual intercourse three or more times a week before pregnancy obtained from the positive items of the Attitudes towards Sexuality in Pregnancy Scale was 4.060 units higher than was that obtained by the participants who had sexual intercourse once a week (*p* = 0.017). The mean score the participants who did not avoid sexual intercourse during pregnancy obtained from the positive items of the Attitudes towards Sexuality in Pregnancy Scale was 6.920 units higher than was that obtained by the participants who avoided it (*p* < 0.001). It was determined that each one-unit increase in pre-pregnancy weight led to a 0.171-unit increase in the score of the positive items of the Attitudes towards Sexuality in Pregnancy Scale (*p* = 0.006). On the other hand, each one-unit increase in acceptance of physical appearance of pregnancy led to a 0.395-unit decrease in the positive items score of the Attitudes towards Sexuality in Pregnancy Scale (*p* = 0.005). Each one-unit increase in weight gain anxiety led to a 0.413-unit decrease in the positive items score of the Attitudes towards Sexuality in Pregnancy Scale (*p* = 0.005). The other variables included in the model had no significant effect on the scores of the positive items of the Attitudes towards Sexuality in Pregnancy Scale (*p* > 0.05) (Table 4).

**Table 4:** Factors affecting attitudes towards sexuality in pregnancy scale scores (*n* = 460)

Variables	Unstandardized coefficient		Standardized coefficient	t	*p*	95% CI	
	B	SE	Beta			Lower	Upper
(Constant)	111.546	7.291		15.298	0.000	97.216	125.875
Education=Bachelor’s degree	6.856	1.861	0.169	3.683	0.000	3.198	10.514
Education=Master’s degree	13.812	3.143	0.178	4.394	0.000	7.635	19.990
Spouse’s education=Bachelor’s degree	4.177	1.776	0.102	2.351	0.019	0.686	7.668
Paying attention to appearance before pregnancy = no	-4.818	2.014	-0.095	-2.393	0.017	-8.776	-0.861
Being knowledgeable about having sexual intercourses during pregnancy = no	-10.395	2.157	-0.206	-4.818	0.000	-14.634	-6.155
Being satisfied with sexual life before pregnancy = yes	11.094	4.732	0.092	2.345	0.019	1.795	20.393
Frequency of sexual intercourses before pregnancy = 3 or more per week	4.060	1.697	0.093	2.393	0.017	0.726	7.395
Abstaining from sexuality during pregnancy = no	6.920	1.622	0.173	4.267	0.000	3.733	10.107
Pre-pregnancy Weight	0.171	0.061	0.107	2.780	0.006	0.050	0.292
Acceptance of Physical Appearance of Pregnancy	-0.395	0.140	-0.124	-2.819	0.005	-0.670	-0.120
Concerns about Weight Gain	-0.413	0.147	-0.126	-2.798	0.005	-0.702	-0.123

F (11, 448) = 21.831. *p* < 0.001; Adj. R2 = 0.333. Variables included in the regression model: having health problems during pregnancy (reference category: yes), nausea and vomiting during pregnancy (reference category: yes), increase in blood pressure level during pregnancy (reference category: yes), increase in blood glucose level during pregnancy (reference category: yes), excessive weight gain during pregnancy (reference category: yes). Employment status (reference category: homemaker), spouse’s employment status (reference category: civil servant), education (reference category: primary school), spouse’s education (reference category: primary school), perceived income (reference category: bad). Family type (education: nuclear), type of pregnancy (reference category: naturally). whether the pregnancy is planned (reference category: planned), miscarriage (reference category: yes), curettage (reference category: yes), paying attention to weight before pregnancy (reference category: yes). paying attention to appearance before pregnancy (reference category: yes), being satisfied with sexual life before pregnancy (reference category: no), frequency of sexual intercourse before pregnancy (reference category: once a week), abstaining from sexuality during pregnancy (reference category: yes), being knowledgeable about having sexual intercourses during pregnancy (reference category: yes), way of perceiving current or potential changes in body image (reference category: positive), being influenced by someone else in perceiving current or potential changes in body image (reference category: yes). Number of pregnancies (reference category: primiparous), pre-pregnancy weight, gestational age, height, age, spouse’s age, duration of marriage, age at first pregnancy, pregnancy (current) weight, physical burdens of pregnancy, concerns about weight gain, acceptance of physical appearance of pregnancy

## Discussion

According to the results of the study, the mean ages of the participants and their spouses were 28.42 ± 5.04 and 31.58 ± 5.42 years, respectively. As reported in the literature, the average age of pregnant women generally ranges between 25 and 30, since it is a period of high fertility and sexual awareness, both physically and psychologically [22, 23]. Thus, the mean age of the pregnant women in the present study is consistent with that in the literature. However, the age distribution ranging from 18 to 45 suggests that differences in maturity and experience brought on by age may have affected the participating pregnant women’s body image, and their knowledge and awareness of sexuality.

Of the participants, 54.1% perceived the change in their body image positively, which is consistent with the literature reporting that pregnant women’s perceptions of their body image are heterogeneous [5, 21, 24]. In a meta-analysis in which feeling of being dissatisfied with body image during pregnancy was investigated indicated that body satisfaction in pregnant women was generally not significantly different from that in non-pregnant women, but individual differences such as cultural factors, social support, education level might be determinants [25]. Similarly, in another study, women with a positive body image displayed significantly more positive attitudes towards sexuality during pregnancy [23], which suggests that the high proportion of the participants who perceived their body image positively in the present study may have influenced their sexual attitudes positively.

In the present study, the majority of the participants prioritized their appearance and paid attention to their weight before pregnancy. However, 11.7% of them were influenced by the opinions of people in their environment about changes in their bodies. Similarly, in a qualitative study conducted in Iran, pregnant women’s body image was shaped by their emotions and feedback they received from people in their environment. Especially, feedback received from their partners played a decisive role in their body image and attitudes towards sexuality [26]. Similarly, a study conducted to determine the relationship between pregnant women’s self-perception, dyadic adjustment, and their attitudes towards sexuality during pregnancy, and to identify the influencing factors, revealed that pregnant women’s perceptions of motherhood and body image were moderate, the quality of their relationships with their partners was good, but their attitudes towards sexuality during pregnancy were negative. Maternal perception and dyadic adjustment are positively correlated with attitudes towards sexuality during pregnancy, while body image is negatively correlated. Maternal perception and dyadic adjustment are also significant determinants of attitudes towards sexuality during pregnancy [22]. Therefore, improving relationship satisfaction and agreement, which influence perception of maternal perception and dyadic adjustment during pregnancy, may strengthen pregnant women’s positive attitudes towards sexuality, which supports the results in the literature on the effect of spousal support on body image and sexual life during pregnancy.

Of the participants, 80.4% were knowledgeable about sexual intercourse during pregnancy. As emphasized in the literature, knowledge about sexuality during pregnancy positively affects sexual attitudes and behaviors [23, 27]. On the other hand, lacking knowledge about sexuality during pregnancy has been reported to increase fear, embarrassment, and sexual avoidance behaviors [28, 29]. From this perspective, the participants’ having high level of knowledge may support them in making conscious and safe choices in their sexual lives. However, 49.3% of the participants abstained from sexual intercourse during pregnancy. As reported in the literature, a decrease in the frequency of sexual intercourse during pregnancy is common and is usually due to physical discomfort, fear of harming the baby, or changes in body image [22, 27]. Of the participants in the present study, 42% experienced health problems during pregnancy, which may have led to a decrease in sexual desire, and avoidance of sexual activity.

The mean score the participants obtained from the ASPS was 123.50 ± 20.06. Considering the possible score range (34–170) and the cut-off point (111.5), the participants’ mean score above the cut-off point indicates that they generally displayed positive attitudes towards sexuality during pregnancy, consistent with the literature [23].

The mean score the participants obtained from the BUMPS was 50.91 ± 11.87, which is consistent with the mean scores indicated in the literature. As reported in a meta-analysis, acceptance of body image during pregnancy is heterogeneous, since some women accept it positively and others accept it negatively [25]. In the present study, the overall body image was moderately positive, which can be interpreted as a finding supporting sexual attitude scores which were above the cutoff point.

The results of the multivariate linear regression analysis revealed that each one-unit increase in the level of acceptance of physical appearance of pregnancy led to a 0.395-unit decrease in the score of the positive items of the Attitudes towards Sexuality in Pregnancy Scale (*p* = 0.005), and that each one-unit increase in weight gain anxiety led to a 0.413-unit decrease in the score of the positive items of the Attitudes towards Sexuality in Pregnancy Scale (*p* = 0.005). This result indicates that changes in pregnant women’s body perception directly affect their sexual attitudes, and as the body image becomes more negative, so do attitudes towards sexuality. This result of the present study is consistent with the results of Aygör and Koçak’s study (2025) indicating that women with a negative genital self-image during pregnancy displayed significantly more negative attitudes towards sexuality [30]. On the other hand, in Kahveci and Cirban Ekrem’s study (2025), as the body perception of pregnant women became more positive, their attitudes towards sexuality became more negative, indicating that body image is not limited to physical appearance alone, and that it is a multidimensional phenomenon closely related to women’s self-perception, sexual identity perception and psychosocial satisfaction level [22].

In the present study’s model, variables such as “educational status,” “being satisfied with pre-pregnancy sexual life,” and “frequency of pre-pregnancy sexual intercourse” significantly affected body image were also addressed, indicating that in the present study, a broader set of predictors were included compared to studies conducted on sexuality during pregnancy, in which the focus was largely on body image. Thus, the results of the present study are expected to contribute to the literature since the study provides a multidimensional analysis of the factors influencing attitudes towards sexuality during pregnancy. Furthermore, given the number of studies in the literature with comprehensive variable sets is limited. This result supports the originality of our study.

### Strengths and limitations

The strength of the present study is that psychosocial factors such as body image, sociodemographic characteristics, and obstetric characteristics that influence attitudes towards sexuality during pregnancy were addressed together. However, the single-center nature of the study limits the generalizability of the results to all pregnant women.

## Conclusion and recommendations

In the present study, it was revealed that the participating pregnant women had moderately positive body image, and that they generally displayed positive attitudes towards sexuality. Increased acceptance of physical appearance of pregnancy and decreased negative perceptions of weight gain are associated with more positive attitudes towards sexuality during pregnancy. Furthermore, the participating pregnant women’s and their partners’ educational levels, and certain pre-pregnancy- and pregnancy-related factors had significant effects on their attitudes towards sexuality during pregnancy. Taken together, these findings suggest that interventions aimed at strengthening pregnant women’s body image may positively influence their attitudes towards sexuality. Therefore, nurses, midwives, and other healthcare professionals involved in the care of pregnant women should be encouraged to identify women’s needs with cultural sensitivity in mind. It is recommended that such professionals plan and implement education and counseling programs focused on enhancing body image and supporting a healthy sexual life during pregnancy.

## Supplementary Information

Supplementary Material 1.

## Data Availability

The datasets used and/or analysed during the current study are available from the corresponding author on reasonable request.
