# Supplementary material for: Effects of pregnant women’s body image on their sexual attitudes
Source: BMC Pregnancy Childbirth. 2026 Mar 3;26:381. doi: 10.1186/s12884-026-08876-x (PMC13064344; doi:10.1186/s12884-026-08876-x)
Supplement: Supplementary file 1 — Supplementary Material 1. [file 12884_2026_8876_MOESM1_ESM.docx]

**Effects of Pregnant Women's Body Image on Their Sexual Attitudes**

**Abstract**

In this descriptive, correlational study, the aim was to investigate the effect of body image on sexual attitudes in pregnant women. The study was conducted with 460 pregnant women who attended the Pregnancy School of a Training and Research Hospital in Turkey between March 2025 and July 2025. Data were collected using the Descriptive Characteristics Form, Body Understanding Measure for Pregnancy Scale (BUMPS), and Attitudes towards Sexuality in Pregnancy Scale (ASPS). Pearson correlation was used to analyze the relationships between continuous variables, and the multiple linear regression analysis was used to determine the factors affecting attitudes towards sexuality using backward elimination. The mean age of the participants was 28.42 ± 5.04 years. The largest proportion held a bachelor’s degree, and a substantial proportion of their spouses also had a bachelor’s degree. The mean score the participants obtained from the ASPS was 123.50±20.06, above the cut-off point (111.5); indicating that the *participating pregnant women’s* (hereafter referred to as “participants”) attitudes towards sexuality were generally positive. The mean score they obtained from the BUMPS score was 50.91±11.87, indicating that they had a moderately positive body image.

The results of the multiple linear regression analysis revealed that the educational status of the participants and their partners, and certain pre-pregnancy and pregnancy-related factors significantly affected the participants’ attitudes towards sexuality during pregnancy. In conclusion, as the participants’ acceptance of their body image increased and their concerns about weight gain decreased, they displayed more positive attitudes towards sexuality. It is recommended that nurses should be encouraged to plan supportive interventions that address pregnant women’s body image and attitudes towards sexuality.

**Keywords:** Body perception; sexual attitude; pregnancy

**Background**

Body image, a person’s internalized self-perception of his or her own body, is multidimensional. Body image encompasses a person's perceptions, thoughts, attitudes, and feelings regarding his or her physical characteristics such as weight, shape, thinness, muscularity, sexual attractiveness, athletic appearance, and functionality (1). During pregnancy, the body's shape, weight, and resulting appearance change, causing the pregnant woman to focus on her own body. Although pregnancy is a normal physiological process, women can experience both physical and psychological problems during this period due to changes in their body image (2).

Changes in a pregnant woman's body begin in the first trimester, but they don't affect their body image, because they develop slowly. As pregnancy progresses, these changes can affect women both positively and negatively. Rapid and intense changes occur in a pregnant woman's body during the second trimester, and these changes can cause the woman to have a different perception of herself and to feel clumsy, awkward, ugly, or unattractive. Due to these changes, a pregnant woman's self-confidence and self-esteem can decrease, which, in turn, negatively affects her body image (3). In the third trimester, these changes reach their peak. Due to the changes experienced throughout pregnancy, some pregnant women perceive their body image positively, while others are dissatisfied with it. In a study in which pregnant women’s self-esteem and body image were compared, their self-esteem was better in the first trimester than was that in the second and third trimesters (4).

Among the factors that influence body image during pregnancy are readiness for motherhood, planned pregnancy, adequate knowledge about pregnancy and birth, socioeconomic conditions, and a woman's perception of her physical characteristics. In their study Küçükkaya et al. (2020) determined that as the woman’ s pregnancy-related body image improved, so did her acceptance of pregnancy, and that her improved acceptance of pregnancy had a positive effect on her body image (5). While some women experience a more comfortable pregnancy without any complications, others experience a more stressful pregnancy. The stress experienced due to weight gain and changes in appearance can cause pregnant women to have a sense of inadequacy and various emotional problems that can lead to crises. This feeling of inadequacy can cause pregnant women to perceive their body image negatively (6). As a result of having this negative perception of body image, many pregnant women may strive to maintain their pre-pregnancy body standards. They may eat unhealthily out of concern for weight gain, leading to significant health problems for both themselves and their babies.

During pregnancy, body image directly affects a woman's attitudes and behaviors towards pregnancy and sexuality. Among pregnancy-related changes are nausea and vomiting, worsened sleep and excretion patterns, breast swelling and tenderness, and skin changes like striae gravidarum and linea nigra, which women struggle to cope with and adapt to. Pregnant women may feel overweight and worry about whether they will regain their former appearance after giving birth. All these factors, in turn, affect a woman's sexual life (7, 8).

Women's sexual functioning, and attitudes towards sexuality vary throughout their married lives, especially during pregnancy. During pregnancy, women and their partners experience a dilemma: on the one hand, they postpone sexual intercourse out of fear of harming the baby, and on the other hand, they consider that sexual intercourse is necessary to maintain a healthy marriage (9, 10). In a study, 88.8% of the participating pregnant women experienced sexual dysfunction, and the rates of sexual dysfunction in the first, second and third trimester were 81.8%, 85.7%, and 91.9%, respectively (11). In Nakic Rados et al.’s study (2015), 79.3% of the women experienced a decrease in the frequency of sexual intercourse compared to their pre-pregnancy period, 41% of them did not engage in sexual intercourse in the last month of pregnancy, and their sexual desire and sexual function decreased in the third trimester (12). In Jamali and Mosalanejad’s study conducted in Iran (2013), 76.2% of the participating pregnant women experienced sexual dysfunction in the third trimester (13). As determined in several other studies, factors such as back pain, weight gain, and dyspnea during the third trimester of pregnancy led to abstinence from sexual activities (12, 14). As determined in many studies, pregnant women's sexuality-related anxiety, beliefs, and values, and their attitudes towards approving sexuality during pregnancy affect their sexual functioning (12, 14, 15, 16, 17).

Displaying a positive attitude towards sexuality during pregnancy can reduce sexual dysfunction (18). In Erbil's study (2019), it was determined that being overweight and obese negatively affected pregnant women’s sexual function, that the pregnant woman's body image had no effect on sexual function, and that duration of pregnancy, number of pregnancies, fewer sexual intercourses, and changes in the partner's sexual attitudes affected sexual function during pregnancy (11). In Kumcağız's study (2012), younger pregnant women had more positive body image than did other pregnant women, but they also exhibited negative attitudes towards sexuality during pregnancy (6). In another study, higher education levels decreased women’s negative attitudes towards sexuality during pregnancy (10). Pregnant women’s and their partners’ negative attitudes towards the physical and psychological changes that occur during pregnancy and their fear of harming the baby discourage them from having sexual intercourse during pregnancy (19, 20).

Since women experience physical, psychological, and sociocultural changes during pregnancy, the effect of their attitudes towards sexual function and sexuality during this period should not be overlooked. Being aware that pregnant women are likely to have negative body image and attitudes towards sexuality can help prevent them from experiencing potential psychological and sexual health problems during this period. Maintaining sexual functions during pregnancy, which affects physical, psychological, sociocultural, and family health, is of great importance in terms of protecting family health. The review of the literature revealed that in no studies, effects of pregnant women's body image on their sexual attitudes were investigated.

Thus, in the current study, answers to the following questions were sought:

1. What is the body image of pregnant women like?

2. What attitudes do pregnant women display towards sexuality?

3. Does the body image of pregnant women affect their attitudes towards sexuality?

4. What are the factors that influence pregnant women's body image and attitudes towards sexuality?

**Method**

**Design and participants**

In the present study, the descriptive and correlational research design was used. The study population consisted of pregnant women who attended the Pregnancy School at a Training and Research Hospital in Turkey. Based on hospital records from the previous year (2024), the known population size was 983 pregnant women. The minimum sample size was calculated as 276 using the sampling method for known population (confidence interval: 95%, margin of error: 0.05). However, considering the possibility of withdrawals and/or losses during the study, it was decided to include a higher number of pregnant women (N=460) in the sample.

**Inclusion criteria**

Being ≥18 years old, volunteering to participate in the study, being fluent in Turkish, being literate, having no diagnosis of a chronic or psychiatric illness, not being diagnosed with a high-risk pregnancy, being primiparous or multiparous, and being in the second or third trimester of pregnancy.

**Research variables**

Independent variables: socio-demographic and obstetric characteristics of the participants, and their body perception,

Dependent variable: Scores obtained from the Attitudes towards Sexuality in Pregnancy Scale (ASPS).

**Data collection**

This single-center study was conducted at a Training and Research Hospital in Turkey. Data were collected from the pregnant women who attended the Pregnancy School between March 2025 and July 2025 after the necessary permissions were obtained. After the women were informed about the purpose and content of the study in collaboration with the hospital, written informed consent was obtained from those who met the inclusion criteria and volunteered to participate. Then, data were collected from them through face-to-face interviews, conducted in a single session, in a quiet, confidential environment, which ensured that the interviews were conducted comfortably, safely, and effectively.

**Data collection tools**

***Descriptive Characteristics Form:*** The form consists of items questioning the participants’ socio-demographic characteristics such as age, education level, employment status, family type and income level, and obstetric characteristics such as desire for pregnancy, and health problems during pregnancy.

***Body Understanding Measure for Pregnancy Scale (BUMPS)***: The BUMPS was developed by Kirk and Preston (2019) (21). The validity study of the Turkish version of the BUMPS was performed by Duman et al. (2023) (1). The BUMPS administered to assess pregnant women's body image during pregnancy consists of 19 items whose responses are rated on a five-point Likert-type scale ranging from 1 to 5, and the following 3 subscales: “Being Satisfied Appearing Pregnant”, “Concerns about Weight Gain”, “Physical Burdens of Pregnancy”. The minimum and maximum possible scores that can be obtained from the BUMPS are 19 and 95, respectively. The higher the score obtained from the “Acceptance of Physical Appearance of Pregnancy” subscale is, the lower the level of body satisfaction during pregnancy is. The higher the scores obtained from the “Concerns about Weight Gain” and “Physical Burdens of Pregnancy” subscales are, the higher the levels of negative perception and concern related to these areas are. The scale consists of positively and negatively keyed statements. While positively keyed statements express undesirable emotions, negatively keyed statements express desired emotions. Items 1, 4, 6, 8, 10, 11, 15, and 19 in the BUMPS are negatively keyed statements and the scores obtained from them are reversed. The scale has no cut-off point. The Cronbach's α reliability coefficient of the BUMPS was calculated as 0.91 in Kirk & Preston’s, and Duman et al.’s studies, and 0.85 in the present study.

***Attitudes towards Sexuality in Pregnancy Scale (ASPS):*** The ASPS developed by Yılmaz Sezer and Şentürk Erenel (10) is used to assess pregnant women’s and their partners’ attitudes towards sexuality during pregnancy. The ASPS consists of 34 items and the following 3 subscales: Anxiety towards Sexual Intercourse during Pregnancy (items 7, 10, 15, 18, 22, 25, 26, 27, 30), Beliefs and Values ​​Towards Sexuality during Pregnancy (items 3, 4, 8, 9, 12, 13, 16, 17, 19, 29), and Approval of Sexuality during Pregnancy (items 1, 2, 5, 6, 11, 14, 20, 21, 23, 24, 28, 31, 32, 33, 34). Items 1, 2, 5, 6, 11, 14, 20, 21, 23, 24, 28, 31, 32, 33, 34 refer to positive attitudes. Items 3, 4, 7, 8, 9, 10, 12, 13, 15, 16, 17, 18, 19, 22, 25, 26, 27, 29, 30 which refer to negative attitudes are reverse scored. The lowest and highest possible scores that can be obtained from the ASPS are 34 and 170, respectively. While the lowest possible scores that can be obtained from the “Anxiety towards Sexual Intercourse during Pregnancy”, “Beliefs and Values ​​Towards Sexuality during Pregnancy” and Approval of Sexuality during Pregnancy subscales are 9, 10, and 15, respectively, the highest possible scores that can be obtained from them are 45, 50, and 75, respectively. The higher the score obtained from the ASPS is, the more positive the respondent’s attitude towards sexuality during pregnancy is. The cut-off point for the ASPS is 111.5. If the score is above the cut-off point, it indicates positive attitude, and if it is below the cut-off point, it indicates negative attitude. The Cronbach's Alpha coefficients of the overall ASPS and its “Anxiety towards Sexual Intercourse”, “Beliefs and Values ​​towards Sexuality during Pregnancy” and “Approval of Sexuality during Pregnancy” subscales were 0.90, 0.85, 0.86 and 0.81, respectively in Yılmaz Sezer and Şentürk Erenel’s study (10), and 0.92, 0.84, 0.90, 0.83, respectively in the present study.

**Statistical analysis**

The data obtained were analyzed using the IBM SPSS (Statistical Package for the Social Sciences) version 23.0. Normality analysis was conducted to determine whether the properties of continuous variables were normally distributed. In this context, the Kolmogorov-Smirnov test was applied, and skewness and kurtosis values, histogram and Q-Q plot graphs, were taken into account. While continuous variables were presented as mean ± standard deviation (Mean±SD), categorical variables were presented as number (n) and percentage (%). Pearson's correlation coefficient was calculated to analyze the relationship between the continuous variables. Multiple linear regression analysis based on the backward elimination method was performed to determine the factors affecting attitudes towards sexuality during pregnancy. p values less than 0.05 were considered significant.

**Ethical considerations**

To conduct the study, ethics committee approval was obtained from the Karamanoğlu Mehmetbey University Health Sciences Scientific Research and Publication Ethics Committee (Decision Date: September 04. 2024, Decision Number: 01-2024/09). Permission to conduct the study in the aforementioned hospital was obtained from the Provincial Health Directorate. Participation in the study was voluntary, and written informed consent was obtained from all the pregnant women who volunteered to participate in the study.

**Results**

The mean age of the participants and their spouses were 28.42±5.04 (min: 18, max: 45) and 31.58±5.42 (min: 20, max: 53) years, respectively. The mean duration of marriage was 5.02±4.39 years (min: 1, max: 25). The mean number of pregnancies was 2.09±1.20 (min: 1, max: 7). The mean number of living children was 0.81±0.93 (min: 0, max: 4). The mean gestational age was 27.98±8.41 (min: 13, max: 40) weeks. The mean age at first pregnancy was 24.52±4.01 (min: 16, max: 40) years. The average pre-pregnancy weight of the participants was 63.89±12.60 (min: 30, max: 110) kg. The average weight during pregnancy (current weight) was 73.35±13.25 (min: 40, max: 124) kg. The average height was 161.85±6.13 (min: 145, max: 183) cm. Of the participants, 73.2% were homemakers and 42.4% had a bachelor's degree. Of their spouses, 38.8% had a bachelor's degree and 50.5% were workers. Of the participants, 71.7% perceived their income level as medium, 90.7% had a nuclear family, 14.1% had experienced curettage, 18% had experienced a miscarriage, 94.1% conceived naturally, 72.4% had planned pregnancies, 80.9% paid attention to their pre-pregnancy appearance, 72.4% paid attention to their pre-pregnancy weight, 42% experienced health problems during pregnancy, 30% experienced nausea and vomiting, 5.9% experienced excessive weight gain, 2.8% experienced increased blood pressure levels, 3.3% experienced increased blood glucose levels, and 8% experienced other health problems (Table 1). The other individual and pregnancy-related characteristics of the participants are presented in Table 1.

Table 1. Participants' individual and pregnancy-related characteristics (n=460)

| **Variables** | **Number (n)** | **Percentage (%)** |
| --- | --- | --- |
| **Participants’ Age (years)** (Mean: 28.42±5.04. Min-Max:18-45) |  |  |
| **Participants’ Spouses’ Age (years)** (Mean: 31.58±5.42. Min-Max:20-53) |  |  |
| **Duration of marriage (years)** (Mean: 5.02±4.39. Min-Max:1-25) |  |  |
| **Number of pregnancies** (Mean: 2.09±1.20. Min-Max:1-7) |  |  |
| **Number of living children** (Mean: 0.81±0.93. Min-Max:0-4) |  |  |
| **Gestational age (weeks)** (Mean: 27.98±8.41. Min-Max:13-40) |  |  |
| **Age at first pregnancy** (Mean: 24.52±4.01. Min-Max:16-40) |  |  |
| **Pre-pregnancy weight (kg)** (Mean: 63.89±12.60. Min-Max:30-110) |  |  |
| **Current weight (kg)** (Mean: 73.35±13.25. Min-Max:40-124) |  |  |
| **Height (cm)** (Mean: 161.85±6.13. Min-Max:145-183) |  |  |
| **Employment status** |  |  |
| Homemaker | 337 | 73.2 |
| Civil servant | 79 | 17.2 |
| Worker | 40 | 8.7 |
| Retired | 4 | 0.9 |
| **Education** |  |  |
| Primary school | 12 | 2.6 |
| Junior high school | 82 | 17.8 |
| Senior high school | 138 | 30.0 |
| Bachelor's degree | 195 | 42.4 |
| Master's degree | 33 | 7.2 |
| **Spouses’ education** |  |  |
| Primary school | 15 | 3.3 |
| Junior high school | 84 | 18.3 |
| Senior high school | 156 | 33.9 |
| Bachelor's degree | 179 | 38.8 |
| Master's degree | 26 | 5.7 |
| **Spouses’ employment status** |  |  |
| Civil servant | 128 | 27.8 |
| Worker | 232 | 50.5 |
| Self-employed | 100 | 21.7 |
| **Perceived income** |  |  |
| Good | 119 | 25.9 |
| Medium | 330 | 71.7 |
| Bad | 11 | 2.4 |
| **Family type** |  |  |
| Nuclear | 417 | 90.7 |
| Extended | 43 | 9.3 |
| **Curettage** |  |  |
| Yes | 65 | 14.1 |
| No | 395 | 85.9 |
| **Miscarriage** |  |  |
| Yes | 83 | 18.0 |
| No | 377 | 82.0 |
| **Type of Pregnancy** |  |  |
| Naturally | 433 | 94.1 |
| Treatment | 27 | 5.9 |
| **Whether the pregnancy was planned** |  |  |
| Planned | 333 | 72.4 |
| Unplanned | 127 | 27.6 |

Table 1. The participants' individual and pregnancy-related characteristics (n=460) (continued)

| **Variables** | **Number (n)** | **Percentage (%)** |
| --- | --- | --- |
| **Paying attention to appearance before pregnancy** |  |  |
| Yes | 372 | 80.9 |
| No | 88 | 19.1 |
| **Paying attention to weight before pregnancy** |  |  |
| Yes | 333 | 72.4 |
| No | 127 | 27.6 |
| **Having health problems during pregnancy** |  |  |
| Yes | 193 | 42.0 |
| No | 267 | 58.0 |
| **Nausea and vomiting** |  |  |
| Yes | 138 | 30.0 |
| No | 322 | 70.0 |
| **Excessive weight gain** |  |  |
| Yes | 27 | 5.9 |
| No | 433 | 94.1 |
| **Increase in blood pressure level** |  |  |
| Yes | 13 | 2.8 |
| No | 447 | 97.2 |
| **Increase in blood glucose level** |  |  |
| Yes | 15 | 3.3 |
| No | 445 | 96.7 |
| **Other health problems** |  |  |
| Yes | 37 | 8.0 |
| No | 423 | 92.0 |
| **The way of perceiving current or potential changes in body image** |  |  |
| Positive | 249 | 54.1 |
| Negative | 105 | 22.8 |
| No idea | 106 | 23.0 |
| **Being influenced by someone else in perceiving current or potential changes in body image** |  |  |
| Yes | 54 | 11.7 |
| No | 406 | 88.3 |
| **Persons influencing their perception of body image** |  |  |
| Friends | 4 | 7.4 |
| Her family | 13 | 24.1 |
| Spouse | 22 | 40.7 |
| Spouse’s family | 1 | 1.9 |
| Social circle | 14 | 25.9 |
| **The way how she was influenced** |  |  |
| Positively | 31 | 57.4 |
| Negatively | 23 | 42.6 |
| **Being knowledgeable about having sexual intercourse during pregnancy** |  |  |
| Yes | 370 | 80.4 |
| No | 90 | 19.6 |
| **Frequency of sexual intercourse before pregnancy** |  |  |
| Once a week | 70 | 15.2 |
| Twice a week | 222 | 48.2 |
| Three or more times a week | 141 | 30.7 |
| Once every two weeks | 27 | 5.9 |
| **Being satisfied with sexual life before pregnancy** |  |  |
| No | 13 | 2.8 |
| Yes | 447 | 97.2 |
| **Avoiding sexuality during pregnancy** |  |  |
| Yes | 227 | 49.3 |
| No | 233 | 50.7 |

Table 2. Mean scores the participants obtained from the scales

| **Scales** | **X** | **±SD** | **Min.** | **Max.** | **Possible score range** | **Cut-off point** |
| --- | --- | --- | --- | --- | --- | --- |
| **Attitudes towards Sexuality in Pregnancy Scale** | 123.50 | ±20.06 | 60 | 168 | 34-170 | 111.5 |
| **Body Understanding Measure for Pregnancy Scale** | 50.91 | ±11.87 | 23 | 87 | 19-95 | - |
| Acceptance of Physical Appearance of Pregnancy | 24.76 | ±6.28 | 9 | 41 | 9-45 | - |
| Concerns about weight gain | 17.63 | ±6.11 | 7 | 35 | 7-35 | - |
| Physical burdens of pregnancy | 8.51 | ±2.43 | 3 | 15 | 3-15 | - |

The mean score the participants obtained from the Attitudes towards Sexuality in Pregnancy Scale was 123.50±20.06. Considering that the possible score range from the scale is 34–170 and the cut-off point is 111.5, the mean score the participants obtained was above the cut-off point (Table 2). The mean scores the participants obtained from the overall Body Understanding Measure for Pregnancy Scale and its “Acceptance of Physical Appearance of Pregnancy”, “Concerns about Weight Gain”, “Physical Burdens of Pregnancy” subscales were 50.91±11.87, 24.76±6.28, 17.63±6.11 and 8.51±2.43, respectively (Table 2).

Table 3. The relationship between the “Attitudes towards Sexuality in Pregnancy Scale”, and “Acceptance of Physical Appearance of Pregnancy”, “Concerns about Weight Gain” and. “Physical Burdens of Pregnancy subscales” (n=460)

| **Variables** | | Attitudes towards Sexuality in Pregnancy Scale |
| --- | --- | --- |
| **Acceptance of Physical Appearance of Pregnancy** | r | -0.297^**^ |
|  | p | 0.000 |
| **Concerns about Weight Gain** | r | -0.287^**^ |
|  | p | 0.000 |
| **Physical Burdens of Pregnancy** | r | -0.212^**^ |
|  | p | 0.000 |

Not: r: Pearson correlation coefficient, *: p<0.05, **: p<0.001

A low-level negative significant correlation was determined between the scores obtained from the “positive items of the Attitudes towards Sexuality in Pregnancy Scale” and the “Acceptance of Physical Appearance of Pregnancy” (r=-0.297, p<0.001), “Concerns about Weight Gain” (r=-0.287, p<0.001) and “Physical Burdens of Pregnancy” (r=-0.212, p<0.001) subscales (Table 3).

Table 4. Factors affecting Attitudes towards Sexuality in Pregnancy Scale scores (n=460)

| **Variables** | **Unstandardized coefficient** | | **Standardized coefficient** | **t** | **p** | **95% CI** | |
| --- | --- | --- | --- | --- | --- | --- | --- |
|  | **B** | **SE** | **Beta** |  |  | **Lower** | **Upper** |
| (Constant) | 111.546 | 7.291 |  | 15.298 | 0.000 | 97.216 | 125.875 |
| Education=Bachelor’s degree | 6.856 | 1.861 | 0.169 | 3.683 | 0.000 | 3.198 | 10.514 |
| Education=Master’s degree | 13.812 | 3.143 | 0.178 | 4.394 | 0.000 | 7.635 | 19.990 |
| Spouse’s education=Bachelor’s degree | 4.177 | 1.776 | 0.102 | 2.351 | 0.019 | .686 | 7.668 |
| Paying attention to appearance before pregnancy = no | -4.818 | 2.014 | -0.095 | -2.393 | 0.017 | -8.776 | -0.861 |
| Being knowledgeable about having sexual intercourses during pregnancy = no | -10.395 | 2.157 | -0.206 | -4.818 | 0.000 | -14.634 | -6.155 |
| Being satisfied with sexual life before pregnancy = yes | 11.094 | 4.732 | 0.092 | 2.345 | 0.019 | 1.795 | 20.393 |
| Frequency of sexual intercourses before pregnancy = 3 or more per week | 4.060 | 1.697 | 0.093 | 2.393 | 0.017 | .726 | 7.395 |
| Abstaining from sexuality during pregnancy = no | 6.920 | 1.622 | 0.173 | 4.267 | 0.000 | 3.733 | 10.107 |
| Pre-pregnancy Weight | 0.171 | 0.061 | 0.107 | 2.780 | 0.006 | 0.050 | 0.292 |
| Acceptance of Physical Appearance of Pregnancy | -0.395 | 0.140 | -0.124 | -2.819 | 0.005 | -0.670 | -0.120 |
| Concerns about Weight Gain | -0.413 | 0.147 | -0.126 | -2.798 | 0.005 | -0.702 | -0.123 |
| Note: F (11, 448) = 21.831. p<0.001; Adj. R2=0.333. Variables included in the regression model: having health problems during pregnancy (reference category: yes), nausea and vomiting during pregnancy (reference category: yes), increase in blood pressure level during pregnancy (reference category: yes), increase in blood glucose level during pregnancy (reference category: yes), excessive weight gain during pregnancy (reference category: yes). Employment status (reference category: homemaker), spouse's employment status (reference category: civil servant), education (reference category: primary school), spouse's education (reference category: primary school), perceived income (reference category: bad). Family type (education: nuclear), type of pregnancy (reference category: naturally). whether the pregnancy is planned (reference category: planned), miscarriage (reference category: yes), curettage (reference category: yes), paying attention to weight before pregnancy (reference category: yes). paying attention to appearance before pregnancy (reference category: yes), being satisfied with sexual life before pregnancy (reference category: no), frequency of sexual intercourse before pregnancy (reference category: once a week), abstaining from sexuality during pregnancy (reference category: yes), being knowledgeable about having sexual intercourses during pregnancy (reference category: yes), way of perceiving current or potential changes in body image (reference category: positive), being influenced by someone else in perceiving current or potential changes in body image (reference category: yes). Number of pregnancies (reference category: primiparous), pre-pregnancy weight, gestational age, height, age, spouse's age, duration of marriage, age at first pregnancy, pregnancy (current) weight, physical burdens of pregnancy, concerns about weight gain, acceptance of physical appearance of pregnancy. | | | | | | | |

In Table 4, the results of the multivariate linear regression analysis conducted to determine the factors influencing attitudes towards sexuality during pregnancy are given. The created model significantly explained attitudes towards sexuality during pregnancy by 33.3% (F (11, 448) = 21.831, p<0.001, Adj. R² = 0.333). The mean scores the participants with Bachelor’s and Master’s degrees obtained from the positive items of the Attitudes towards Sexuality in Pregnancy Scale were 6.856 and 13.812 units higher than were those obtained by the participants who were primary school graduates, respectively (p<0.001). The mean score the participants whose spouses had a Bachelor’s degree obtained from the positive items of the Attitudes towards Sexuality in Pregnancy Scale was 4.177 units higher than was that obtained by the participants whose spouses were primary school graduates (p=0.019). The mean score the participants who did not pay attention to their appearance before pregnancy obtained from the positive items of the Attitudes towards Sexuality in Pregnancy Scale was 4.818 units lower than was that obtained by the participants who paid attention to their appearance before pregnancy (p=0.017). The mean score the participants who were not knowledgeable about sexuality during pregnancy obtained from the positive items of the Attitudes towards Sexuality in Pregnancy Scale was 10.395 units lower than was that obtained by the participants who were not knowledgeable (p<0.001). The mean score the participants who were satisfied with their sexual life before pregnancy obtained from the positive items of the Attitudes towards Sexuality in Pregnancy Scale was 11.094 units higher than was that obtained by the participants who were not (p=0.019). The mean score the participants who had sexual intercourse three or more times a week before pregnancy obtained from the positive items of the Attitudes towards Sexuality in Pregnancy Scale was 4.060 units higher than was that obtained by the participants who had sexual intercourse once a week (p=0.017). The mean score the participants who did not avoid sexual intercourse during pregnancy obtained from the positive items of the Attitudes towards Sexuality in Pregnancy Scale was 6.920 units higher than was that obtained by the participants who avoided it (p<0.001). It was determined that each one-unit increase in pre-pregnancy weight led to a 0.171-unit increase in the score of the positive items of the Attitudes towards Sexuality in Pregnancy Scale (p=0.006). On the other hand, each one-unit increase in acceptance of physical appearance of pregnancy led to a 0.395-unit decrease in the positive items score of the Attitudes towards Sexuality in Pregnancy Scale (p=0.005). Each one-unit increase in weight gain anxiety led to a 0.413-unit decrease in the positive items score of the Attitudes towards Sexuality in Pregnancy Scale (p=0.005). The other variables included in the model had no significant effect on the scores of the positive items of the Attitudes towards Sexuality in Pregnancy Scale (p>0.05) (Table 4).

**Discussion**

According to the results of the study, the mean ages of the participants and their spouses were 28.42±5.04 and 31.58±5.42 years, respectively. As reported in the literature, the average age of pregnant women generally ranges between 25 and 30, since it is a period of high fertility and sexual awareness, both physically and psychologically (22, 23). Thus, the mean age of the pregnant women in the present study is consistent with that in the literature. However, the age distribution ranging from 18 to 45 suggests that differences in maturity and experience brought on by age may have affected the participating pregnant women's body image, and their knowledge and awareness of sexuality.

Of the participants, 54.1% perceived the change in their body image positively, which is consistent with the literature reporting that pregnant women's perceptions of their body image are heterogeneous (5, 21, 24). In a meta-analysis in which feeling of being dissatisfied with body image during pregnancy was investigated indicated that body satisfaction in pregnant women was generally not significantly different from that in non-pregnant women, but individual differences such as cultural factors, social support, education level might be determinants (25). Similarly, in another study, women with a positive body image displayed significantly more positive attitudes towards sexuality during pregnancy (23), which suggests that the high proportion of the participants who perceived their body image positively in the present study may have influenced their sexual attitudes positively.

In the present study, the majority of the participants prioritized their appearance and paid attention to their weight before pregnancy. However, 11.7% of them were influenced by the opinions of people in their environment about changes in their bodies. Similarly, in a qualitative study conducted in Iran, pregnant women's body image was shaped by their emotions and feedback they received from people in their environment. Especially, feedback received from their partners played a decisive role in their body image and attitudes towards sexuality (26). Similarly, a study conducted to determine the relationship between pregnant women's self-perception, dyadic adjustment, and their attitudes towards sexuality during pregnancy, and to identify the influencing factors, revealed that pregnant women's perceptions of motherhood and body image were moderate, the quality of their relationships with their partners was good, but their attitudes towards sexuality during pregnancy were negative. Maternal perception and dyadic adjustment are positively correlated with attitudes towards sexuality during pregnancy, while body image is negatively correlated. Maternal perception and dyadic adjustment are also significant determinants of attitudes towards sexuality during pregnancy (22). Therefore, improving relationship satisfaction and agreement, which influence perception of maternal perception and dyadic adjustment during pregnancy, may strengthen pregnant women's positive attitudes towards sexuality, which supports the results in the literature on the effect of spousal support on body image and sexual life during pregnancy.

Of the participants, 80.4% were knowledgeable about sexual intercourse during pregnancy. As emphasized in the literature, knowledge about sexuality during pregnancy positively affects sexual attitudes and behaviors (23, 27). On the other hand, lacking knowledge about sexuality during pregnancy has been reported to increase fear, embarrassment, and sexual avoidance behaviors (28, 29). From this perspective, the participants’ having high level of knowledge may support them in making conscious and safe choices in their sexual lives. However, 49.3% of the participants abstained from sexual intercourse during pregnancy. As reported in the literature, a decrease in the frequency of sexual intercourse during pregnancy is common and is usually due to physical discomfort, fear of harming the baby, or changes in body image (22, 27). Of the participants in the present study, 42% experienced health problems during pregnancy, which may have led to a decrease in sexual desire, and avoidance of sexual activity.

The mean score the participants obtained from the ASPS was 123.50±20.06. Considering the possible score range (34–170) and the cut-off point (111.5), the participants' mean score above the cut-off point indicates that they generally displayed positive attitudes towards sexuality during pregnancy, consistent with the literature (23).

The mean score the participants obtained from the BUMPS was 50.91±11.87, which is consistent with the mean scores indicated in the literature. As reported in a meta-analysis, acceptance of body image during pregnancy is heterogeneous, since some women accept it positively and others accept it negatively (25). In the present study, the overall body image was moderately positive, which can be interpreted as a finding supporting sexual attitude scores which were above the cutoff point.

The results of the multivariate linear regression analysis revealed that each one-unit increase in the level of acceptance of physical appearance of pregnancy led to a 0.395-unit decrease in the score of the positive items of the Attitudes towards Sexuality in Pregnancy Scale (p=0.005), and that each one-unit increase in weight gain anxiety led to a 0.413-unit decrease in the score of the positive items of the Attitudes towards Sexuality in Pregnancy Scale (p=0.005). This result indicates that changes in pregnant women’s body perception directly affect their sexual attitudes, and as the body image becomes more negative, so do attitudes towards sexuality. This result of the present study is consistent with the results of Aygör and Koçak’s study (2025) indicating that women with a negative genital self-image during pregnancy displayed significantly more negative attitudes towards sexuality (30). On the other hand, in Kahveci and Cirban Ekrem’s study (2025), as the body perception of pregnant women became more positive, their attitudes towards sexuality became more negative, indicating that body image is not limited to physical appearance alone, and that it is a multidimensional phenomenon closely related to women's self-perception, sexual identity perception and psychosocial satisfaction level (22).

In the present study’s model, variables such as "educational status," "being satisfied with pre-pregnancy sexual life," and "frequency of pre-pregnancy sexual intercourse" significantly affected body image were also addressed, indicating that in the present study, a broader set of predictors were included compared to studies conducted on sexuality during pregnancy, in which the focus was largely on body image. Thus, the results of the present study are expected to contribute to the literature since the study provides a multidimensional analysis of the factors influencing attitudes towards sexuality during pregnancy. Furthermore, given the number of studies in the literature with comprehensive variable sets is limited. This result supports the originality of our study.

**Strengths and limitations**

The strength of the present study is that psychosocial factors such as body image, sociodemographic characteristics, and obstetric characteristics that influence attitudes towards sexuality during pregnancy were addressed together. However, the single-center nature of the study limits the generalizability of the results to all pregnant women.

**Conclusion and recommendations**

In the present study, it was revealed that the participating pregnant women had moderately positive body image, and that they generally displayed positive attitudes towards sexuality. Increased acceptance of physical appearance of pregnancy and decreased negative perceptions of weight gain are associated with more positive attitudes towards sexuality during pregnancy. Furthermore, the participating pregnant women’s and their partners’ educational levels, and certain pre-pregnancy- and pregnancy-related factors had significant effects on their attitudes towards sexuality during pregnancy. Taken together, these findings suggest that interventions aimed at strengthening pregnant women’s body image may positively influence their attitudes towards sexuality. Therefore, nurses, midwives, and other healthcare professionals involved in the care of pregnant women should be encouraged to identify women's needs with cultural sensitivity in mind. It is recommended that such professionals plan and implement education and counseling programs focused on enhancing body image and supporting a healthy sexual life during pregnancy.

**References**

1. Duman M, Doğan Yüksekol Ö, Timur Taşhan S. The Turkish version of Body Understanding Measure for Pregnancy Scale: validity and reliability study. J Health Sci. 2023;32(2):168-174.
2. Gür EY, Pasinlioğlu T. Determining status of perception of the pregnant women regarding body image during pregnancy. J Midwifery Health Sci. 2020;3(2):71-81.
3. Taşkın L, Kukulu K. Kadın sağlığına giriş. In: Taşkın L, editor. Doğum ve kadın sağlığı hemşireliği. Ankara: Sistem Ofset Matbaacılık; 2011. p.117.
4. İnanır S, Çakmak B, Nacar MC, Güler AE, İnanır A. Body image perception and self-esteem during pregnancy. Int J Womens Health Reprod Sci. 2015;3(4):196–200.
5. Küçükkaya B, Altan Sarıkaya N, Kahyaglu Süt H, Öz S. The relation between body perception related to the weight gain during pregnancy and acceptance of pregnancy. JAREN. 2020;6(3):426-32.
6. Kumcağız H. Pregnant women, body image and self-esteem according to the examination of some of the variables. Int J Human Sci. 2012;9(2):691-703.
7. Çetin SA, Aslan E. Kadın cinsel sağlığı ve kadına yönelik cinsel şiddet. In: Kızılkaya BN, editor. Hemşire ve ebelere yönelik kadın sağlığı ve hastalıkları. İstanbul: Nobel Tıp Kitabevi; 2015. p.161-193.
8. Kul Uçtu A, Bekmezci H, Özerdogan N. Sexuality during pregnancy. Gümüşhane Univ J Health Sci. 2017;6(3):171-175.
9. Arıca SG, Alkan S, Bali S, Mansuroğlu E, Özer C. The effect of pregnancy over sexual life. J Turk Fam Physician. 2014;2(3):19–24.
10. Yılmaz Sezer N, Şentürk Erenel A. Development of an attitude scale toward sexuality during pregnancy. J Sex Marital Ther. 2021;47(5):492-507.
11. Erbil N. The relationship between sexual function, body image and body mass index among pregnant women. Int J Caring Sci. 2019;12(2):925-936.
12. Nakic Rados S, Vranes HS, Sunjic M. Sexuality during pregnancy: what is important for sexual satisfaction in expectant fathers? J Sex Marital Ther. 2015;41(4):282–293.
13. Jamali S, Mosalanejad L. Sexual dysfunction in Iranian pregnant women. Iran J Reprod Med. 2013;11(6):479-486.
14. Navidian A, Rigi SN, Soltani P. Effects of group sexual counseling on the traditional perceptions and attitudes of Iranian pregnant women. Int J Womens Health. 2016;8:203-211.
15. Galazka I, Drosdzol Cop A, Naworska B, Czajkowska M, Skrzypulec-Plinta V. Changes in sexual function during pregnancy. J Sex Med. 2015;12(2):445–454.
16. Bilen Sadi Z, Aksu H. The sexual life of partners in pregnancy and examination of affecting factors. Anadolu Hemşirelik ve Sağlık Bilimleri Dergisi. 2016;19(2):128–138.
17. Leite CC, Masochini RG, Cunha AN, Paese MC, Barros CR, Jezus SV, Cavalcante PP. Sexuality during pregnancy: perceptions of pregnant women in an educational group. Sci Electron Arch. 2020;13(4):76–85.
18. Abouzari-Gazafroodi K, Najafi F, Kazemnejad E, Rahnama P, Montazeri A. Demographic and obstetric factors affecting women’s sexual functioning during pregnancy. Reprod Health. 2015;12:72.
19. Sossah L. Sexual behavior during pregnancy: A descriptive correlational study among pregnant women. Eur J Res Med Sci. 2014;2(1):16–27.
20. Yeniel AO, Petri E. Pregnancy, childbirth, and sexual function: perceptions and facts. Int Urogynecol J. 2014;25(1):5–14.
21. Kirk E, Preston C. Development and validation of the Body Understanding Measure for Pregnancy Scale (BUMPS) and its role in antenatal attachment. Psychol Assess. 2019;31(9):1092–1106.
22. Kahveci M, Cirban Ekrem E. The determination of the relationship between pregnant women's self-perception and dyadic adjustment in relation to sexual attitudes, and identification of influencing factors. Unika Sag Bil Derg. 2025;5(1):42–59.
23. Öcalan D, Toker S, Kılıç Doğan E, Alparslan Ö. Is there a relationship between pregnant women's sexual attitudes and body image? Afr J Reprod Health. 2024;28(11):56–67.
24. Karasin FB, Yilmaz T. Psychometric properties of the Turkish version of BUMPs and its relationship with prenatal attachment. J Reprod Infant Psychol. 2025;43(1):62–75. doi:10.1080/02646838.2023.2217718
25. Crossland AE, Munns L, Kirk E, Preston CEJ. Comparing body image dissatisfaction between pregnant women and non-pregnant women: a systematic review and meta-analysis. BMC Pregnancy Childbirth. 2023;23:709.
26. Sohrabi Z, Kazemi A, Farajzadegan Z, Janighorban M. Body perception in pregnant women: a qualitative study. BMC Pregnancy Childbirth. 2023;23:165.
27. Dinc H, Kizilkaya Beji N. The effect of pregnancy on women’s sexual function, body image and pelvic floor functions. Kuwait Med J. 2018;50(1):17–23.
28. Yuvarlan E, Beydağ KD. Attitudes of pregnants towards sexuality in pregnancy and affecting factors. BANU J Health Sci Res. 2024;6(1):21–31.
29. Öztürk Altınayak S, Hür S, Yurtçiçek Eren S, Arslan B, Artan M. Evaluation of pregnant women's attitudes toward sexuality. JOWHEN. 2024;10(3):138–149.
30. Aygör H, Koçak V. The association of genital self-image during pregnancy with the attitude toward sexuality. J Obstet Gynaecol Res. 2025;51(4):e16291. doi:10.1111/jog.16291

**Declarations**

**Ethics approval and consent to participate:** Approval was granted by the Karamanoğlu Mehmetbey University Health Sciences Scientific Research and Publication Ethics Committee (Decision Date: September 04. 2024, Decision Number: 01-2024/09). Permission to conduct the study was also obtained from the Provincial Health Directorate. Written informed consent to participate was obtained from all participants in accordance with the Declaration of Helsinki.

**Consent for publication:** Not applicable.

**Availability of data and materials:** The datasets used and/or analysed during the current study are available from the corresponding author on reasonable request.

**Competing interests:** The authors declare that they have no competing interests.

**Source of Funding:** The authors declare that no funds, grants, or other support were received during the preparation of this manuscript.

**Author Contributions:** All authors read and approved the final manuscript. MG contributed to conceptualization, methodology, data collection, data organization, analysis, and writing (original draft); YŞ contributed to conceptualization, data collection, analysis, and writing (review and editing).

**Acknowledgements:** We would like to extend our gratitude to all participants.
